# Nutrient reference values for bioactives: new approaches needed? A conference report

**DOI:** 10.1007/s00394-013-0503-0

**Published:** 2013-03-01

**Authors:** Hans Konrad Biesalski, John W. Erdman, John Hathcock, Kathleen Ellwood, Stephen Beatty, Elizabeth Johnson, Roberto Marchioli, Lotte Lauritzen, Harry B. Rice, Andrew Shao, James C. Griffiths

**Affiliations:** 1 University of Hohenheim, Stuttgart, Germany; 2 University of Illinois at Urbana-Champaign, Urbana, IL USA; 3 Consultant for Council for Responsible Nutrition, Washington, DC USA; 4 College of Southern Maryland, La Plata, MD USA; 5 Waterford Institute of Technology, Waterford, Ireland; 6 Tufts University, Boston, MA USA; 7 Consorzio Mario Negri Sud, Santa Maria Imbaro, Italy; 8 University of Copenhagen, Copenhagen, Denmark; 9 Global Organization for EPA and DHA Omega-3s, Salt Lake City, UT USA; 10 Herbalife Ltd., Torrance, CA USA; 11 Council for Responsible Nutrition, Washington, DC USA

**Keywords:** Nutrient reference values, Non-essential nutrients, Adequate intake, Lutein, Zeaxanthin, Meso-zeaxanthin, n-3 Long-chain polyunsaturated fatty acids

## Abstract

Nutrients can be classified as either “essential” or “non-essential,” the latter are also termed bioactive substances. Whereas the absence of essential nutrients from the diet results in overt deficiency often times with moderate to severe physiological decrements, the absence of bioactive substances from the diet results in suboptimal health. Nutrient reference values are set by Codex Alimentarius and regulatory bodies in many countries, mostly for essential nutrients with recommended daily intakes. The IOM in the United States has defined a set of four DRIs that, when data are appropriate, include an EAR, a RDA that is derived from the EAR, an AI for nutrients without appropriate data to identify an EAR, and an UL. From the RDA, the United States derives a labeling value called the DV, which applies to older children and most adults. In Codex, the equivalents of the DVs are the NRVs to be used in calculating percentage values on food labels. Nothing in the IOM documents specifies that labeling values can be set only for what have been defined to date as essential nutrients. Indeed, the US Food and Drug Administration sets a labeling value for dietary fiber based on the IOM AI for this ingredient. This conference explores the definitions, concepts, and data on two of the best examples of bioactive substances that, perhaps, should have NRVs: lutein and zeaxanthin, and n-3 long-chain polyunsaturated fatty acids.

## Background, definitions, and principles

It can be argued that the first official public health dietary guidance originated with the British Merchant Seaman’s Act in 1835, which suggested lime or lemon juice for sailors to prevent what we now know as scurvy. Since then, guidelines for healthy food patterns have been refined and are still important nutrition education tools for health professionals and consumers. Every 5 years (since 1980) in the United States, the food pattern-based Dietary Guidelines for Americans (DGA) are published [1]. In 1941, the National Research Council released the first set of Recommended Dietary Allowances (RDAs) for energy, protein, and eight vitamins and minerals. The RDAs are quantitative values that are translated to food patterns for the DGA and many federal food programs and thus began the nutrient-based guidelines in the United States. The 10th edition of the RDAs was published in 1989 containing numerical recommendations for 27 “essential” nutrients (out of 49 total nutrients).

Beginning in the mid-1990s, the Dietary Reference Intakes (DRIs) were created by the Food and Nutrition Board of the US National Academies’ Institute of Medicine (IOM) to broaden the concept of RDAs beyond “alleviation of nutrient deficiency diseases” to embrace promotion of good health. The DRIs include RDAs (and Recommended Nutrient Intakes (RNIs) in Canada), as well as estimated average requirements (EARs), adequate intakes (AIs), acceptable macronutrient distribution ranges (AMDRs), and tolerable upper intake levels (ULs). The amount of clinical evidence necessary for establishing RDAs is substantial, and there are well-established procedures for deriving RDAs. For example, a depletion/repletion trial with hospitalized human volunteers was the foundation for the establishment of the EAR, RDA, and UL for vitamin C [2]. Reference values like RDAs and AIs are used to ensure sufficient intakes of essential nutrients. Estimation of the reference range is based, in many cases, on observational data linked to deficiency symptoms or intakes by healthy groups. By adding a safety range, it is argued that the intake will be sufficient and safe.

Foods contain a variety of macronutrients and non-essential components. Macronutrients can be essential and/or be oxidized as fuels and provide carbon skeletons and amino groups for endogenous synthesis of body constituents. Emerging evidence suggests that both the traditional essential and some non-essential portions of foods may provide specific health benefits. As in the case for essential nutrients, some of the non-essential food components cannot be made by the body. But if they have health benefits and are considered to be part of a healthy diet, they could be considered as bioactive food components. In contrast to essential nutrients, non-essential bioactive food components may not have any clearly described clinical deficiency symptoms when their intake is inadequate. Vitamins A and C must be obtained from the diet to prevent deficiency diseases, but what about non-provitamin A producing carotenoids such at lutein and lycopene or the functionally active long-chain derivatives of essential fatty acids, such as eicosapentaenoic acid (EPA) and docosahexaenoic acid (DHA)? The weight of emerging experimental evidence suggests that these and other food bioactive substances, including dietary fibers and some polyphenols, may contribute to health.

In contrast to essential nutrients, it may not be possible to carry out experiments to prove cause-effect relationships for non-essential food components. Indeed, studies of vitamins during the last century resulted in the detection and identification of these essential compounds. In contrast, there are inherent difficulties with constructing human trials for non-essential food components, whether provided as pure substances, in foods or from food extracts. These include lack of validated biomarkers, blinding of test subjects, and availability of funding for the studies. A further limitation, especially for bulky ingredients such as dietary fiber, is the near impossibility of devising a placebo control. One could argue that more evidence should be necessary when it comes to making dietary recommendations for non-essential food components, but the amount of evidence that might be practically collected may be substantially less in comparison with that which is expected for essential nutrients. On the other hand, so-called non-essential food components may provide health benefits in ways not yet identified. Epidemiological studies show some evidence that a diet rich in lutein may be protective against age-related macular degeneration (AMD). Could it be that AMD is a symptom of lutein deficiency? Evidence also exists that indicate a number of dietary components may have protective effects on multi-factorial lifestyle diseases such as coronary heart disease (CHD) and cancer, for example, the cardioprotective effects of a diet rich in marine-derived n-3 long-chain polyunsaturated fatty acids (n-3 LCPUFA) or the protective effects against prostate cancer from a diet rich in tomato products or lycopene. However, this relationship should not be used to imply that prostate cancer is a symptom of lycopene deficiency. Thus far, the decreased risks of AMD, CHD, and prostate cancer can only be said to be associated with diets rich in lutein, n-3 LCPUFA or lycopene, respectively. Thus, in the case of non-essential nutrients, there may be diet related, but not single nutrient related, “deficiency” disorders, and recommendations on how to prevent these “deficiency” disorders may be warranted.

Accordingly, a new paradigm that establishes recommendations for non-essential bioactive food components may be necessary; one that may differ from the traditional DRI approach. Some suggest that the “totality of the evidence” should be sufficient to drive public health messages about non-essential bioactive food components. In this report, we first review the DRI framework that has been in place for over 15 years. Second, we address potential mechanisms for “accreditation” of bioactive food components and discuss issues regarding design of studies, risk/benefit ratios, lack of biomarkers, genetic variability within the population, challenges in research funding, and the consequences of the possible negative effects of not taking any action. Finally, we apply the concept to two examples: lutein and related compounds, and the n-3 LCPUFAs EPA and DHA.

## The development of nutrient reference values

The long-term goal of developing a new nutrient paradigm is to provide consumers with appropriate public health guidance about healthy food choices for both traditional nutrients and bioactive food components. The Codex Committee on Nutrition and Foods for Special Dietary Uses (CCNFSDU) is in the process of developing daily nutrient reference values (NRVs) for the purpose of nutrition labeling. The development and sanction of the NRV should provide protection and reassurance to both the consumer and the food industry, that is, protection of the consumer against claims that a product “contains” a specific ingredient even if the amount is trivial by any nutritional standard and protection of the food industry by defining a “safe harbor” for product composition that will allow effective, truthful, and non-misleading communication with the consumer. In line with this new concept, the European Food Safety Authority (EFSA) recently concluded that exposure to ß-carotene from its use as a food additive and as a food supplement at a level <15 mg/day does not contribute to adverse health effects in the general population, including heavy smokers [3].

Emerging data (from in vitro studies, in vivo studies in animals and some studies in humans) have shown that non-essential food components may be beneficial to health. Whether the data can be used to document any causality in health-related effects of bioactive components needs further investigation. The scientific data must demonstrate consistent results that show that the health impact can be attributed to the food component of interest. However, most of the available data are based on observational studies and consequently on food or dietary patterns containing high or low amounts of a particular bioactive component. Furthermore, many bioactive components are “biomarkers” for a healthy dietary pattern. Vitamin E is a good marker for edible plant-derived oils and seedlings, and zeaxanthin is a biomarker for orange-colored foods (egg yolk, corn, orange, melon, paprika). As long as we do not have a clear clinical symptom of an inadequate intake of a non-essential nutrient, we need to refer to a food or dietary pattern for a reference value.

The AI is a recommended average daily intake level based on observed or experimentally determined approximations or estimates of nutrient intake by a group (or groups) of apparently healthy people that are assumed to be adequate in nutrition status. There is nothing in the IOM definition specifying that the nutrient must be essential to have an assigned AI value. So-called non-essential nutrients include those oxidized as fuels and those that provide carbon skeletons and amino groups for endogenous synthesis of body constituents. They can consist of some food components that may have health benefits and are considered as part of a healthy diet and may also have a significant impact on health.

In the example of fiber (the DRI term “total fiber” means the combination of dietary fibers and functional fibers), there were enough data demonstrating the potential health benefits of fibers to establish an AI, but not enough to establish an EAR, and from it a RDA. A large body of experimental data acquired since the early 1990s has demonstrated blood cholesterol lowering effects of dietary fibers, which has been supported by epidemiological evidence showing correlation between increased intake of high fiber foods and reduction in risk of CHD. Prospective cohort studies have suggested that diets high in fiber-rich foods decrease the risk for hypertension, a risk factor for CHD. An issue with observational studies examining the effects of dietary fiber is that it is not possible to distinguish between the effects of dietary fiber per se and fiber-rich foods that contain many other food components. Moreover, foods high in fiber are generally low in fat, saturated fat and cholesterol, and high in phytochemicals, all of which are associated with reduced risks of certain chronic diseases. Thus, isolating fiber as a single factor is difficult and must be evaluated in the context of the total dietary pattern. Some investigators have specifically analyzed diets for dietary fiber, and others have used indicators of dietary fiber intake such as cereals, vegetables, fruits, whole grains, or legumes. Despite these differences in assessing fiber intake, the preponderance of the evidence on dietary fiber and CHD risk based on epidemiological, clinical, and mechanistic data was strong enough to set a recommended level of intake (an AI).

In the case of β-carotene and other carotenoids, a DRI was not established because potential diet-disease effects could be due to other substances found in carotenoid-rich foods or to behaviors associated with high fruit and vegetable consumption, for example, regular physical exercise. This potential for misattribution illustrates the importance of understanding the specific role of a nutrient (essential or non-essential) in a food matrix or dietary pattern. Large prospective studies have shown beneficial effects of high carotenoid containing fruit and vegetable consumption with respect to chronic diseases. However, the nutrients/food components responsible for the effects are difficult to ascertain due to multiple nutrient interactions that would need to be isolated for study and to the possible substitution of nutrients known to increase risk for certain chronic diseases, for example, saturated fats. If a dietary pattern can be related to a single non-essential nutrient with respect to a disease risk or disease-related marker, this relationship may serve as a marker to establish a NRV.

It can be difficult to test the effects of individual food components on chronic disease risk. Challenges in obtaining valid, reproducible, and reliable data are numerous but they need to be overcome. Biomarkers for health effects should be related to specific diseases as intermediary endpoints or validated surrogate endpoints of disease risk. The established disease-related surrogate endpoints recognized by the US Food and Drug Administration (FDA) are few. They are as follows: total/low-density lipoprotein (LDL) cholesterol, blood pressure for CHD; polyps for colon/rectal cancer; blood sugar level and insulin resistance for diabetes; bone mineral density for osteoporosis; and mild cognitive impairment for dementia. Large randomized controlled trials (RCTs) play a critical role in establishing the relationship between intake of nutrients (essential/non-essential) and risk of chronic diseases; however, is the RCT design appropriate, and indeed the sole method, to understand the role of whole foods or food constituents in chronic disease prevention? Finally, what is the potential harm in making an evidence-based public health recommendation using data from large prospective cohort studies when there is a lack of large RCTs versus the potential harm of not making any recommendation at all?

## Example: lutein and related compounds

Lutein is a non-provitamin A carotenoid found in green, leafy vegetables and brightly colored fruits. Zeaxanthin is also of dietary origin, mainly derived from corn and corn products. A third related carotenoid, meso-zeaxanthin, is not normally found in a conventional diet and is generated in the retina after lutein isomerization [4]. Challenges inherent in the separation and quantification of meso-zeaxanthin have resulted in a paucity of data on the content of this carotenoid in foodstuffs and have rendered the study of tissue concentrations of this compound problematic. As a consequence, the few studies that have investigated meso-zeaxanthin may have been disproportionately influential in the ongoing debate about its origin.

Lutein, zeaxanthin, and meso-zeaxanthin all accumulate in the central retina where they are collectively known as macular pigment (MP) [5]. Lutein and zeaxanthin are distributed ubiquitously in body tissues, but tend to be concentrated and the dominant carotenoids in central nervous tissues. Along with meso-zeaxanthin, lutein and zeaxanthin are the sole MP carotenoids, where they exist in approximately 500-fold higher concentrations than in other body tissues. Each of these three compounds exhibits a regional dominance, with meso-zeaxanthin, lutein, and zeaxanthin being the dominant carotenoids at the epicenter, mid-periphery, and periphery of the macula, respectively. The MP protects the eye from damage due to short wave length (blue) light and has a strong antioxidant activity. Indeed, data from animal studies give strong evidence that the three carotenoids protect photoreceptors against oxidative injury [6]. There is a growing and evidence-based consensus that MP is important for optimal visual performance, because of its blue light-filtering properties and consequential attenuation of chromatic aberration, veiling luminance, and blue haze. It has also been hypothesized that MP may protect against AMD because of the same optical properties and the antioxidant capacity of the macular carotenoids.

## Carotenoids and visual performance and macular degeneration

Several studies have shown beneficial effects of carotenoids on the progression of AMD [7]. The promising data of the Age-Related Eye Disease Study (AREDS) were confirmed by a recent Cochrane analysis [8]. The authors concluded that people with AMD may experience less progression of the disease as a result of antioxidant vitamin and mineral supplementation. Based on morphological and biochemical data, Loughman and colleagues carried out numerous studies to elucidate which carotenoid is of greatest importance for the protective effect on AMD [9]. Furthermore, they investigated the effect of carotenoids on visual performance [10]. The observational study assessed whether macular pigment optical density (MPOD) is associated with visual performance. One hundred forty-two young healthy subjects were recruited. MPOD and visual performance were assessed by psychophysical tests including best-corrected visual acuity (BCVA), mesopic and photopic contrast sensitivity, glare sensitivity, and photostress recovery time (PRT). Measures of central visual function, including BCVA and contrast sensitivity, were found to be positively associated with MPOD (*p* < 0.05, for all).

RCTs have shown that supplementation with carotenoids (10.6 mg meso-zeaxanthin, 5.9 mg lutein, 1.6 mg zeaxanthin) results in an increase in their concentration in serum as well as in the macula without any adverse effects on either liver or renal function [11, 12]. Several studies were performed to elucidate whether one or more carotenoids detected within the macula are of importance for the MOPD. Supplementation with carotenoids has been shown to result in a typical central peak in the MP only in supplements that contain meso-zeaxanthin [9]. In this RCT, Loughman and colleagues investigated changes in MPOD and visual performance following supplementation with different macular carotenoid formulations: (1) 20 mg lutein and 2 mg zeaxanthin; (2) 10 mg lutein, 2 mg zeaxanthin, and 10 mg meso-zeaxanthin; and (3) placebo. At 3 and 6 months, a statistically significant increase in MPOD was found at all eccentricities (other than the most peripheral 3° location) in the group that got all three carotenoids (*P* < 0.05 for all), whereas no significant increase in MPOD was demonstrable at any eccentricity for subjects that got only lutein + zeaxanthin or placebo. Statistically significant improvements in visual performance measures, including visual acuity and contrast sensitivity with and without glare, were also observed only in those who got meso-zeaxanthin, whereas there was only significant improved mesopic contrast sensitivity at one spatial frequency by 6 months for the lutein + zeaxanthin group and no improvements in any parameters of visual performance for subjects supplemented with placebo. Thus, these data show that all three carotenoids are needed to form the macular pigment. In addition, supplementation has a beneficial effect on visual performance and contrast sensitivity. The carotenoid intake in this recent study may serve as reference range with respect to safety and efficacy.

## Carotenoids and the brain

Lutein is also the dominant carotenoid in human brain tissue. While a variety of evidence supports a role for lutein in eye health, less is available on a relationship between lutein and cognitive function. From observational studies, there is evidence that older adults consuming the highest amounts of green leafy vegetables and cruciferous vegetables, which are both rich sources of lutein, had slower cognitive decline than those consuming the lowest amounts. Several studies at Tufts University also found that lutein status is related to better cognitive function in older adults [13]. A significant relationship was found between serum levels of lutein and cognitive function in a population-based study which looked at biological, psychological, and social factors that play a role in longevity and survival of the oldest old [14]. In the Health, Aging, and Body Composition Study (Health ABC), Renzi and colleagues found a significant correlation between MP density and the mini mental state examination, a global measure of cognition [15]. Lutein and zeaxanthin in the eye of rhesus monkeys have been shown to be significantly related to lutein and zeaxanthin levels in the brain and MP and can therefore be used as a biomarker of lutein and zeaxanthin in primate brain tissue [16]. This finding provides a great advantage as macular lutein and zeaxanthin can now be measured by a non-invasive technique. Postmortem lutein levels in brain tissue have also been found to be significantly related to antemortem measures of global cognitive function, executive function, and dementia severity after adjusting for age, gender, education, hypertension, and diabetes [14]. Lastly, in a double-blinded RCT in older adults supplemented with lutein, alone or in combination with DHA, Johnson and colleagues reported that verbal fluency scores improved significantly with the DHA, with lutein, as well as with the combined treatment groups [17]. Memory scores and rate of learning improved significantly in the combined treatment group, whose subjects also displayed a trend toward more efficient learning. These exploratory findings suggest that lutein supplementation may have cognitive benefit for older adults. Taking all of these observations into consideration, the idea that lutein can influence neural function in older adults is certainly plausible.

## n-3 Long-chain polyunsaturated fatty acids

The n-3 LCPUFAs perform essential functions in the body in processes such as male reproduction and child development. Most experts estimate that the n-3 LCPUFA requirement is fulfilled at intakes of alpha-linolenic acid (ALA) from vegetable oils of around 0.5–1 % of the energy intake. n-3 LCPUFA intake has been shown to affect health, most notably, the risk of CHD and symptoms of rheumatoid arthritis as well as infant visual acuity and atopic risk [18–20]. In addition, n-3 LCPUFA has also been described to have a potential beneficial impact on other degenerative diseases, for example, chronic renal diseases, neurological diseases, and diseases of the eye and the respiratory tract. These health effects seem to be exerted mainly by EPA and DHA. The clinical effect of n-3 LCPUFA on rheumatoid arthritis has been shown only at doses >2.5 g/day, and the effect on visual maturation and atrophy seems to occur only in the perinatal period [18]. Current evidence indicates that n-3 LCPUFA may also affect mood, behavior, and overall immune function, but the evidence does not allow recommendations regarding an exact dose. There is more convincing evidence on the quantitative needs in adults to prevent CHD.

With respect to the cardioprotective effects of n-3 LCPUFA, evidence is available from laboratory and observational studies, as well as RCTs, with various outcomes. n-3 LCPUFA has been shown to affect a myriad of molecular pathways, including alteration of physical and chemical properties of cellular membranes, direct interaction with and modulation of membrane channels and proteins, regulation of gene expression, and conversion of n-3 LCPUFA to bioactive metabolites and signalling molecules, which may all provide plausible biological explanations for the observed effects. RCTs have shown that n-3 LCPUFA supplementation in humans lowers plasma triacylglycerol (TAG), thrombosis, resting heart rate, and blood pressure and might also improve myocardial filling and efficiency. Additional mechanisms include lowering inflammation and improving vascular function while experimental studies demonstrate direct and indirect antiarrhythmic effects [21]. Observational studies consistently suggest long-term dietary intake of n-3 LCPUFA from fish has protective effects on cardiovascular disease when compared with no or very low intake [22]. Large-scale RCTs in patients with previous myocardial infarction and heart failure also suggest a significant benefit on cardiac mortality. However, evidence produced by a recent RCT of n-3 LCPUFA supplementation to prevent atrial fibrillation onset or recurrence in patients with diabetes mellitus who have experienced a myocardial infarction was, however, disappointing [23]. Differences in experimental clinical settings and methodological limitations of recent studies make it difficult to interpret these recent findings. Indeed, data from the recent Alpha Omega Trial indicate that the effect of n-3 LCPUFA is over-shadowed by the use of statins [24]. Overall, current data provide discordant evidence that n-3 LCPUFAs are bioactive compounds that reduce risk of cardiac death. On the other hand, sound evidence supports the health benefit of a regular dietary intake of n-3 LCPUFA, and there is evidence from RCTs to support the beneficial effects on the mentioned cardiovascular risk markers.

Major dietary sources of the n-3 LCPUFA are fatty fish and other seafood. A meta-analysis of data from 29 prospective cohort studies indicates that maximal CHD prevention occurs at a fish intake of around 50 g/day [21]. Furthermore, a separate meta-analysis calculated that the incidence of CHD decreases 6 % per 15 g/day increment in fish intake [25]. The effect on plasma TAG appears to be linear from 1 to 7 g/day n-3 LCPUFA, whereas the anti-thrombotic effect requires doses >4 g/day. The effects on blood pressure and heart rhythm have been shown to occur within the range of typical dietary intakes and to satiate at around 0.75–1 g/day. Depending on the fish species, an intake of 1 g/day n-3 LCPUFA is achievable at daily intakes of 50 g fish and thus fits well with the observational data.

DHA is the dominant n-3 LCPUFA in tissues, and although it can be formed endogenously from ALA, conversion is very low and requires double action of the key limiting enzyme. Thus, it seems difficult to provide an ALA-based diet that raises DHA status as effectively as preformed DHA via the diet, and studies indicate that high intake of ALA may even lead to a decrease in DHA status. The combined level of DHA and EPA in erythrocytes (the Omega-3 Index, Ω3I) has been proposed as a stable proxy measure of status and an optimal way to assess need [26]. Research indicates high CHD risk at Ω3I of <4 %, and the optimal level appears to be >8 % [27]. To reach an Ω3I of 8 %, US CHD patients have been shown to require fish oil supplementation in addition to a fish intake of >2 servings/week [28]. Tissue n-3 LCPUFA levels are affected by polymorphism in fatty acid desaturase encoding genes and by gender, and it may also depend on other dietary aspects. An Ω3I of 8 % was found for moderate fish intakes (around 0.6 g/day n-3 LCPUFA) in lactating women, but data also indicate that daily fish oil supplementation resulted in higher Ω3I than a similar habitual intake from fish, indicating that intake frequency may play a role.

Most observational studies and meta-analyses of data from prospective cohort studies have not been able to show a beneficial effect of n-3 LCPUFA of vegetable origin. However, one prospective cohort study found that ALA intake was associated with reduced CHD risk and the correlation was most pronounced at n-3 LCPUFA intakes <0.1 g/day [29]. Furthermore, a recent meta-analysis of vegetable oil RCTs showed ALA-containing oils reduced risk, whereas pure linolenic acid (LA) oils increased the risk of CHD events [30]. It has been suggested that the need for n-3 LCPUFA is increased by high intake of LA, but observational studies have not been able to show that the effect of n-3 LCPUFA is modified by LA intake [29, 31, 32].

There is consensus among numerous authoritative and regulatory bodies around the world that intake of EPA and DHA is associated with potential health benefits; however, there is inconsistent or missing guidance on ULs for these fatty acids. According to the Codex Guidelines on Nutrition Labelling, the establishment of general population NRVs should take into account ULs established by recognized authoritative scientific bodies [33]. Evaluations from the last couple of years have all concluded that there is insufficient evidence to establish an UL for n-3 LCPUFAs. The following is a brief history on ULs for EPA and DHA.

- Based on a 1989 report, in 1997, the US FDA declared that intakes of EPA and DHA from menhaden oil up to 3 g/day are safe for the general population [34]. The primary reason for the 3 g limit was concern about bleeding. The authors of the 1989 Mitre Corporation report wrote, “An increase in bleeding time is the only prominent health effect observed in humans that has been firmly established as a consequence of fish oil ingestion. This effect has been reported anecdotally in the Eskimo population and consistently observed in studies of healthy human subjects with a daily intake of 3 g of n-3 fatty acids. The magnitude of the effect at this low dose is not a cause for alarm, but a lack of systematic dose–response data precludes prediction of the severity of the effect at higher daily intakes” [35]. Note that more recent reports have indicated much higher levels of n-3 LCPUFA intake without any bleeding issues.
- In its 2005 report on DRIs for dietary fats, the IOM indicated that there were insufficient data to support establishing an UL for EPA and DHA [36].
- In 2009, the German Federal Institute for Risk Assessment (BfR) evaluated EPA and DHA and recommended that no more than 1.5 g/day n-3 LCPUFA from all sources should be consumed and that food not typically containing fat (e.g., water-based beverages) should not be enriched with n-3 LCPUFAs. While selected studies evaluating several health-based endpoints were summarized in the opinion, the basis for the limit of 1.5 g/day was not elucidated [37].
- In 2011, the Norwegian Scientific Committee for Food Safety (VKM) evaluated EPA and DHA and indicated that it was not possible to identify clear adverse effects associated with EPA and DHA for the purpose of setting ULs [38].
- In 2012, EFSA published its scientific opinion related to ULs for n-3 LCPUFAs with the conclusion that the available data were insufficient to establish an UL for n-3 LCPUFAs [39].
- Also in 2012, Spherix Consulting completed its hazard characterization commissioned by the Global Organization for EPA and DHA Omega-3s (GOED) including a range of safety endpoints and adverse effects. No studies were identified that are appropriate to define specific intake levels or intake/response relationships that can be used to define an UL for the investigated effects [40].

## Challenges in establishing upper limits of intake

Originally applied by the IOM to establish ULs, nutrient risk assessment can be used to identify ULs for bioactive substances. An important modification to the classic nutrient risk assessment model is needed, however. By definition, the establishment of an UL value depends on the selection of a no observed adverse effect level (NOAEL) or a lowest observed adverse effect level. For many bioactive substances (lutein, lycopene, coenzyme Q10) and some essential nutrients (vitamin B12 and the amino acids), no hazard has been identified and thus no NOAEL. Therefore, by definition, an UL cannot be established for these substances. The absence of an UL (or some kind of equivalent guidance level to prevent excessive intakes) has been misinterpreted by some to mean that little or no safety data exist on these substances and has led to some overly restrictive and arbitrary policies in some countries.

In 2006, the Food and Agriculture Organization/World Health Organization (FAO/WHO) published the highest observed intake (HOI) approach to nutrient risk assessment. Briefly, the HOI approach is used to establish guidance levels for those nutrients for which no toxicity has been observed. It involves selection of the highest dose tested that can be confidently concluded as safe. While the method has yet to be formally applied, a version of this approach has been applied and published repeatedly [41–43].

Some of the questions the CCNFSDU has been addressing during the eight-step process of establishing NRVs include: which nutrients should be assigned NRVs, whether NRVs should be assigned for macronutrients (e.g., protein), whether there should be more than one NRV per nutrient (e.g., for specific outcomes), and what data or criteria should serve as the basis for selection of NRVs. However, to date, the committee has not fully addressed the critical issue of the impact of the UL intake on the selection of NRVs. For most micronutrients and bioactive food components, recommended intake levels (on which the daily NRV is based) fall far below the UL by several-fold, up to and above an order of magnitude. However, there are examples from individual countries (e.g., the United States, Canada) in which the authoritative body, such as the IOM, has established a RDA that is perilously close to (in the case of zinc for children) or even exceeds (in the case of magnesium in children) the UL. If used as a basis for Codex NRVs, whether for nutrients or bioactive substances, such policy has the potential to cause widespread confusion (as it has in individual countries) among nutrition policy makers and regulators charged with establishing regulatory maximums.

It is therefore critical that ULs (or HOIs observed) for essential micronutrients and bioactive food components be established based on risk assessment, and that these values be given due account when establishing NRVs, to avoid as much as possible the scenario of a NRV being too close or exceeding the UL. Establishing the UL or HOI prior to the NRV can give policy makers added comfort that the chosen values for labeling are also safe for the general population, and it can also help facilitate necessary research on the benefits of higher intakes of these substances, since it provides practical guidance for both researchers and institutional review boards.
